# The Moderating Role of Sports Attitudes in the Association Between Attitudes Toward Violence and Sexual Harassment

**DOI:** 10.3390/bs16050656

**Published:** 2026-04-26

**Authors:** Emirhan Kan, Muhammet Talha Han, Luís Branquinho, Bekir Erhan Orhan, Pedro Forte, José E. Teixeira, Ricardo Ferraz, Muhammet Emin Ketim

**Affiliations:** 1 Physical Education and Sports, Faculty of Sport Sciences, Atatürk University, Erzurum 25240, Türkiye; 2 Physical Education and Sports, Faculty of Hasan Doğan Sport Sciences, Karabük University, Karabük 78000, Türkiye; 3 Biosciences School of Elvas, Polytechnic University of Portalegre, 7300-110 Portalegre, Portugal; 4 Life Quality Research Center (LQRC-CIEQV), 2001-964 Santarém, Portugal; 5 Research Center in Sports Sciences, Health Sciences and Human Development, 6201-001 Covilhã, Portugal; ricardompferraz@gmail.com; 6 Centro de Investigação do Instituto Superior de Ciência Educativas (CI-ISCE), 4560-547 Penafiel, Portugal; 7 Faculty of Sport Sciences, Istanbul Aydın University, Istanbul 34295, Türkiye; 8 Department of Sports, Higher Institute of Educational Sciences of the Douro, 4560-708 Penafiel, Portugal; 9 Research Centre for Active Living and Wellbeing (Livewell), Instituto Politécnico de Bragança, 5300-253 Bragança, Portugal; 10 Department of Sports Sciences, Instituto Politécnico de Bragança, 5300-252 Bragança, Portugal; 11 Department of Sports Sciences, Polytechnic of Cávado and Ave, 4750-810 Guimarães, Portugal; 12 SPRINT—Sport Physical Activity and Health Research & Innovation Center, 6300-559 Guarda, Portugal; 13 Department of Sport Sciences, University of Beira Interior, 6201-001 Covilhã, Portugal

**Keywords:** sexual harassment, attitudes toward violence, attitudes toward sport, moderation

## Abstract

This study examined whether attitude toward sport moderates the association between attitude toward violence and attitude toward sexual harassment among university students. Based on evidence that harassment-supportive beliefs are embedded within broader violence-supportive orientations, sport-related values were tested as a conditional factor. A cross-sectional correlational design was used with 350 undergraduates (45.1% female; M_age = 21.81, SD = 2.57) from a public university in Türkiye. Participants completed validated measures of attitudes toward sexual harassment, violence, and sport. Moderation analysis was conducted using Hayes’ PROCESS Macro (Model 1) with 5000 bootstrap resamples and HC3 standard errors. Gender was included as a control variable; male participants reported significantly higher tolerance toward sexual harassment (β = 7.258, *p* < 0.001). Attitude toward violence was positively associated with attitude toward sexual harassment (B = 0.271, *p* < 0.001). Attitude toward sport showed a small negative main effect (B = −0.199, *p* < 0.001) and significantly moderated this association (B = −0.010, *p* = 0.0008). The model explained 26.06% of the variance (R^2^ = 0.261, F (4, 345) = 33.607, *p* < 0.001). The association weakened at higher sport attitude levels but remained significant, indicating a pattern of conditional attenuation.

## 1. Introduction

Sexual harassment continues to represent a significant social and public health concern across diverse cultural contexts. Despite increasing public awareness and institutional efforts aimed at prevention, harassment-related experiences remain widespread and socially consequential. Recent national evidence illustrates the persistence of this problem. Findings from the large-scale #MeToo 2024 national study, conducted with 3383 U.S. adults using a probability-based sampling framework, indicate that 82% of women and 42% of men reported experiencing at least one form of sexual victimization in their lifetime, with one in four participants reporting harassment within the past year (Raj et al., 2024). These figures underscore the continuing normalization of harassment-related experiences within contemporary societies. Because attitudes shape how individuals interpret, justify, and respond to social behaviors, understanding attitudes toward sexual harassment is essential for addressing the broader normative structures that sustain such behaviors (Trottier et al., 2025).

In social psychology, attitudes are conceptualized as relatively enduring evaluative tendencies toward objects, behaviors, or social phenomena (Eagly & Chaiken, 1993). They integrate cognitive beliefs, affective reactions, and behavioral predispositions and play a central role in shaping moral judgment and action (Ajzen, 1991). Accordingly, attitudes toward sexual harassment reflect the extent to which individuals morally evaluate, legitimize, or condemn harassment-related behaviors. Although sexual harassment is widely acknowledged as unacceptable, legitimizing beliefs and minimization narratives persist across contexts. For instance, recent European data indicate that substantial proportions of respondents consider certain forms of harassment “somewhat acceptable” and believe that women exaggerate reports of abuse (EIGE, 2024). Such findings illustrate that harassment-related attitudes are embedded within broader evaluative systems rather than isolated judgments.

A substantial body of research suggests that attitudes toward sexual harassment are closely aligned with more general violence-supportive belief structures. Meta-analytic evidence demonstrates that gender role beliefs, sexism, and rape myth acceptance strongly predict societal perceptions of sexual violence (Trottier et al., 2025). The “continuum of sexual violence” framework further proposes that seemingly minor acts of harassment and more severe forms of violence share common gendered power dynamics (Kelly, 1987; Flynn et al., 2021). Empirical findings at institutional and societal levels similarly indicate that tolerance toward violence contributes to climates that normalize harassment (Berkowitz et al., 2022; Nowrouzi-Kia et al., 2025). Collectively, these perspectives suggest that support for harassment-related attitudes may co-occur with, and be reinforced by, broader violence-supportive orientations.

At the same time, increasing attention has been given to the socializing role of sport contexts. Engagement in organized sport has been associated with prosocial development, enhanced empathy, and, in certain contexts, lower acceptance of aggression (Shields & Bredemeier, 1995; Rutten et al., 2011; Wang et al., 2025). However, these effects are not uniform. The normative climate of the sport environment plays a decisive role in shaping outcomes (Zhang & Deng, 2024; Xu et al., 2025). Supportive coaching practices and prosocial peer norms have been linked to reduced violent behaviors (Nichols, 2007; Sherry, 2010; Kammerer et al., 2021), whereas environments characterized by dominance hierarchies and hegemonic masculinity may reinforce aggression and coercion (Forbes et al., 2006). These mixed findings indicate that sport is not inherently protective; rather, its influence depends on the value orientations it cultivates.

It is important to distinguish among three conceptually related but distinct constructs that have frequently been conflated in prior research: sport participation, sport climate, and attitudes toward sport. Sport participation refers to behavioral engagement in organized or recreational athletic activity and captures the frequency and type of physical involvement. Sport climate denotes the contextual and normative environment of a given sport setting—including coaching style, peer norms, and institutional culture—that may facilitate or constrain certain attitudes and behaviors. Attitudes toward sport, by contrast, reflect the degree to which individuals endorse internalized value orientations associated with sport, such as fairness, rule adherence, cooperation, and mutual respect (Koçak, 2014; Shields & Bredemeier, 1995; Kavussanu, 2008). These three constructs are empirically and theoretically separable: a person may participate in sport without necessarily internalizing its prosocial values, and a supportive sport climate may promote such values to varying degrees depending on individual receptivity. The present study focuses exclusively on the third construct—attitudes toward sport as internalized value orientations—and does not assume any direct effect of sport participation or sport climate.

Sport has been conceptualized as a moral and normative context in which value systems related to fairness and respect are internalized and expressed (Boardley & Kavussanu, 2007). From a socialization perspective, such value orientations may develop through observational learning and reinforcement processes (Bandura, 1977) and subsequently function as relatively stable evaluative frameworks guiding moral judgments (Ajzen, 1991). Consistent with Social Learning Theory (Bandura, 1977), individuals who are repeatedly exposed to sport-related norms emphasizing fairness and respect may internalize these as guiding evaluative standards that condition how broader belief systems—including violence-supportive attitudes—are organized and expressed.

The scale used in the present study to operationalize attitudes toward sport (Koçak, 2014) encompasses three conceptually relevant dimensions: (1) ethical–moral values, reflecting beliefs about fair play, rule compliance, and respect for opponents; (2) social interaction, capturing orientations toward cooperation, teamwork, and peer solidarity within sport; and (3) competition, representing value orientations toward achievement, perseverance, and performance striving. Critically, these dimensions address normative value content rather than behavioral frequency or environmental context. As such, the scale captures the extent to which sport-related principles have been internalized as guiding evaluative standards—consistent with attitudinal conceptualizations in social psychology (Eagly & Chaiken, 1993; Ajzen, 1991). It should be noted that this instrument does not assess actual sport environments or moral climates, and findings should therefore be interpreted in terms of individual value orientations rather than structural features of sport contexts.

Rather than exerting a simple direct influence on harassment-related attitudes, sport-related value orientations may operate at a more structural level within individuals’ belief systems. Because attitudes toward sexual harassment are embedded within broader violence-supportive orientations (Trottier et al., 2025), endorsement of sport-related values emphasizing fairness and respect may attenuate the alignment between general violence-supportive attitudes and harassment-supportive attitudes. Specifically, individuals who strongly endorse ethical–moral sport values may apply these normative standards as an evaluative filter when forming judgments about interpersonal conduct, thereby weakening the tendency for violence-supportive attitudes to extend into acceptance of sexual harassment. This mechanism is structural rather than behavioral: it does not require actual sport participation but reflects how internalized value orientations organize belief systems. In other words, sport-related attitudes may function not as a primary predictor of harassment-related beliefs but as a contextual factor that shapes how violence-related evaluations translate into harassment-related judgments. Such a moderating perspective is consistent with conceptualizations of moderation in which the strength of an association varies as a function of a third variable (Hayes, 2017).

Despite the growing literature on violence-supportive beliefs and harassment attitudes, limited attention has been directed toward examining whether sport-related attitudinal orientations condition the relationship between these constructs. Existing research has primarily focused on the behavioral and developmental outcomes of sport participation—such as aggression regulation, prosocial conduct, and moral development—rather than examining how value-based orientations toward sport may shape the structural alignment of violence-related belief systems (Nichols, 2007; Rutten et al., 2011; Sherry, 2010; Kammerer et al., 2021; Wang et al., 2025). To date, limited empirical attention has been directed toward testing sport-related attitudinal orientations as conditional factors within violence-related attitudinal frameworks. Examining this potential moderating mechanism within a university population is particularly relevant, as young adulthood represents a critical period for the consolidation of social and moral attitudes.

Although the theoretical framework of the present study is grounded in general social psychological principles, attitudes toward violence are inherently embedded within broader sociocultural contexts. The present research was conducted in Türkiye, a sociocultural context in which traditional gender norms, patriarchal value structures, and social attitudes toward violence may shape how harassment-related beliefs are formed and expressed. Research indicates that gender-based violence and harassment remain prevalent concerns within Turkish society, and that sport participation is a significant domain of socialization for university-aged youth (Sakallı-Uğurlu et al., 2010). Given the well-documented gender differences in harassment-related attitudes—with men consistently reporting higher tolerance than women across cultural contexts (Trottier et al., 2025; Sakallı-Uğurlu et al., 2010)—gender was included in the present model as a control variable to ensure that the primary moderation effect is not confounded by gender-related variance. Therefore, the findings should be interpreted within this specific sociocultural framework.

Accordingly, the present study aims to examine whether attitude toward sport moderates the association between attitude toward violence and attitude toward sexual harassment. Based on the theoretical considerations outlined above, the following hypothesis was proposed:

**H1:**  *Attitude toward sport moderates the positive relationship between attitude toward violence and attitude toward sexual harassment; specifically, this association is significantly attenuated as positive sport attitudes increase.*

**Figure 1:**
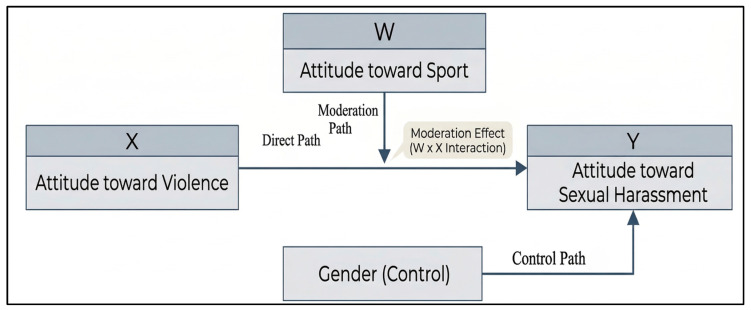
Conceptual Model of the Moderation Effect of Sport Attitude.

The conceptual model presented in Figure 1 outlines the hypothesized interactions and control mechanisms among the primary variables:

*Independent Variable (X—Attitude toward Violence):* This serves as the primary predictor in the model. According to the hypothesis, a positive attitude toward violence is expected to positively correlate with a higher acceptance of or positive attitude toward sexual harassment.

*Dependent Variable (Y—Attitude toward Sexual Harassment):* This is the outcome variable that the study seeks to explain through the interaction of violence and sport attitudes.

*Moderating Variable (W—Attitude toward Sport):* This variable acts as a “modifier” that changes the strength of the relationship between X and Y. Under H1, it is predicted that as an individual’s positive attitude toward sport increases, the predictive power of violent attitudes over sexual harassment attitudes will significantly diminish.

*Control Variable (Gender):* To ensure the statistical robustness of the findings and to isolate the specific effects of the main variables, gender is included in the model to account for potential confounding variance in the dependent variable.

## 2. Materials and Methods

### 2.1. Research Design

This study was conducted using a cross-sectional correlational research design aimed at examining the direction and strength of the relationships among the variables. Cross-sectional correlational designs are widely used to investigate patterns of covariation among variables and to model structural relationships without experimental manipulation (Christensen et al., 2014; Creswell & Creswell, 2017). In this study, regression-based moderation analysis was applied to examine the relationship between attitudes toward violence and attitudes toward sexual harassment and to determine whether attitudes toward sport play a moderating role in this relationship. The analyses were performed using Model 1 of Hayes’ PROCESS macro, which is commonly employed to test moderation models (Hayes, 2017). This analytical approach is appropriate for assessing whether the relationship between the independent variable and the dependent variable varies as a function of the level of a third variable.

### 2.2. Population and Sample

The participants of this study consisted of undergraduate students enrolled in various faculties at Atatürk University in Türkiye. In line with the purpose of the study, a convenience sampling method was employed, based on the voluntary participation of undergraduate students within a single university setting. This approach was selected due to its practical accessibility within the institutional context (Creswell & Creswell, 2017). Accordingly, being enrolled in an undergraduate program at Atatürk University and voluntarily agreeing to participate in the study were determined as the inclusion criteria.

A power analysis based on F-tests was conducted using the G*Power v3.1 program (F tests; Linear multiple regression: Fixed model, R^2^ increase; A priori: Compute required sample size—given α, power, and effect size). In the analysis, the effect size was set to f^2^ = 0.05, α err prob = 0.05, Power (1 − β err prob) = 0.95, Number of tested predictors = 1, and Total number of predictors = 3. Based on these parameters, the minimum required sample size was calculated as Total sample size = 262 (Faul et al., 2009). The final sample consisted of N = 350 participants, exceeding the minimum requirement. Therefore, the sample was considered sufficient to test the moderating role of attitudes toward sport in the relationship between attitudes toward violence and attitudes toward sexual harassment.

As shown in Table 1, of the total 350 students who participated in the study, 45.1% (n = 158) were female, and 54.9% (n = 192) were male. The mean age of the participants was calculated as 21.81 (SD = 2.57). These findings indicate that the research sample has a balanced structure in terms of gender distribution and that the participants fall within the young adult age group.

### 2.3. Data Collection Instruments

#### 2.3.1. Attitudes Toward Sexual Harassment Scale

The scale was originally developed based on the Sexual Harassment Tolerance Scale (Lott et al., 1982) and later revised with additional items (Sakallı-Uğurlu et al., 2010). The Turkish adaptation was conducted by Sakallı-Uğurlu et al. (2010). The scale employs a six-point Likert-type response format (1 = *Strongly Disagree* to 6 = *Strongly Agree*) and comprises three subscales: (1) *viewing sexual harassment as a result of women’s provocative behavior*, (2) *perceiving sexual harassment as natural flirtation*, and (3) *viewing sexual harassment as a serious problem*—the third subscale consists of reverse-coded items. Higher total scores indicate greater tolerance toward sexual harassment (i.e., more accepting attitudes). The Cronbach’s alpha coefficient reported in the original study was 0.79. This scale was preferred because it directly measures individuals’ general evaluative tendencies toward sexual harassment as a social behavior, consistent with the present study’s focus on the integrity of belief systems rather than myth endorsement. The Turkish adaptation has demonstrated acceptable psychometric properties in previous studies (Sakallı-Uğurlu et al., 2010), supporting its cultural appropriateness for the current sample. An example item from the scale is: “I do not see any problem with engaging in frequent physical contact with someone of the opposite sex even in the absence of a close friendship.”

#### 2.3.2. Attitudes Toward Violence Scale

This scale was developed by Davidson and Canivez (2012) and adapted into Turkish by Akın et al. (2016). The scale consists of 17 items rated on a seven-point Likert-type response format and comprises three subscales: *crime and war*, *corporal punishment*, and *partner violence*. Higher scores indicate greater acceptance of violence-related attitudes. The results of the confirmatory factor analysis conducted for the Turkish adaptation indicate that the model fit is at an acceptable level (Akın et al., 2016). An example item from the scale is: “It is acceptable for a spouse to slap their partner if they are humiliated or belittled.”

#### 2.3.3. Attitudes Toward Sport Scale

This scale was developed by Koçak (2014) to measure university students’ attitudes toward sport. The scale consists of 25 items rated on a five-point Likert-type response format and encompasses three subscales: *ethical–moral values*, *competition*, and *social dimension*. These subscales collectively capture the extent to which sport-related normative principles—including fair play, achievement striving, and social cooperation—have been internalized as value orientations, consistent with attitudinal conceptualizations in social psychology (Eagly & Chaiken, 1993; Ajzen, 1991). An example item from the scale is: “Engaging in sports helps to cope with stress.” In the present study, a composite score was computed across all items, reflecting an overall orientation toward sport-related values. It is important to note that this scale does not assess behavioral participation frequency, actual sport environments, or moral climates within specific sport contexts. Accordingly, findings should be interpreted in terms of individually held value orientations rather than structural or contextual features of sport settings. Total scores range from 22 to 110, with higher scores reflecting more positive value orientations toward sport. The construct validity of the scale has been supported in previous studies (Koçak, 2014), and reliability and validity were re-examined in the present sample; relevant results are reported in the Validity–Reliability section.

#### 2.3.4. Personal Information Form

In the study, a personal information form prepared by the researchers was used to determine the demographic characteristics of the participants. This form includes questions regarding the participants’ gender and age. Participants were asked to indicate their gender and age.

According to the confirmatory factor analysis results presented in Table 2, χ^2^/df was found to be 3.55 for Attitude toward Violence, 3.18 for Attitude toward Sexual Harassment, and 3.38 for Attitude toward Sport. A χ^2^/df ratio between 3 and 5 indicates acceptable model fit (Kline, 2023). The Goodness-of-Fit Index values were calculated as 0.89 for Attitude toward Violence, 0.86 for Attitude toward Sexual Harassment, and 0.85 for Attitude toward Sport. The Root Mean Square Error of Approximation value was 0.08 for all three models. RMSEA values of 0.08 and below indicate acceptable fit (Kline, 2023). These findings indicate acceptable, though not uniformly strong, model fit across the three measurement models. The borderline nature of certain indices—particularly for ATSH (χ^2^/df = 3.18, GFI = 0.86, RMSEA = 0.08) and ATS (χ^2^/df = 3.38, GFI = 0.85, RMSEA = 0.08)—should be considered when interpreting the psychometric adequacy of these instruments.

Internal consistency reliability was assessed using Cronbach’s alpha coefficient. The values obtained were 0.841 for Attitude toward Violence, 0.70 for Attitude toward Sexual Harassment, and 0.932 for Attitude toward Sport. Values of 0.70 and above are considered good, whereas values between 0.60 and 0.70 are regarded as acceptable levels of internal consistency (George & Mallery, 2003).

The normality assumption of the dataset was examined based on skewness and kurtosis values. For Attitude toward Violence, skewness was calculated as 0.449 and kurtosis as 0.356; for Attitude toward Sexual Harassment, skewness was 0.632, and kurtosis was −0.089; and for Attitude toward Sport, skewness was −0.532 and kurtosis was −0.402. Skewness and kurtosis values within the range of ±1 to ±2 indicate that the assumption of normal distribution is satisfied (Hair et al., 2014).

When the obtained goodness-of-fit indices, internal consistency coefficients, and skewness and kurtosis values are evaluated together, it can be concluded that the measurement instruments used in this study are sufficiently appropriate in terms of validity and reliability for the research sample. These findings indicate that the scales used possess adequate psychometric properties to measure the constructs examined within the scope of the study and that the dataset meets the necessary assumptions for subsequent statistical analyses.

### 2.4. Ethics Approval

Ethical approval for this study was obtained from the Atatürk University Faculty of Sports Sciences Ethics Committee. The research was reviewed and approved on 23 May 2025, under protocol number 2500167224.

### 2.5. Data Collection Procedure

The research was conducted in accordance with the ethical standards established for scientific studies involving human participants, and the required approval was obtained from the relevant institutional ethics committee prior to the initiation of the data collection process. Participants were informed about the purpose of the study, the voluntary nature of participation, and the principles of confidentiality. Written informed consent was obtained from all participants.

The data were collected in groups through self-report questionnaires administered using the paper-and-pencil method in classroom settings and similar institutional environments. In order to minimize social desirability bias, participation was ensured to be fully anonymous; no identifying information was requested from the participants, and the researchers were not present in the environment during item-level responses. Participants completed the questionnaires individually in a quiet setting and submitted their responses without including any personally identifiable information. The administration process took approximately 10–15 min.

### 2.6. Data Analysis

Data analyses were conducted using the IBM SPSS Statistics 27 software package and Hayes’ PROCESS Macro add-on (Model 1). Prior to the analyses, the dataset was examined in terms of missing data, outliers, and the normality assumption. Z-scores, Mahalanobis distances, as well as skewness and kurtosis values were inspected, and it was determined that the normality assumption was satisfied. In addition, the internal consistency coefficients of the scales used in this study were recalculated, and Cronbach’s alpha coefficients ranged from acceptable to high levels of internal consistency.

Before conducting the regression analyses, model assumptions were tested. Tolerance and Variance Inflation Factor (VIF) values were examined to assess the presence of multicollinearity. Tolerance values of 1.00 and VIF values of 1.00 for the independent variables indicated the absence of a multicollinearity problem and that the assumptions required for regression analysis were met.

In line with the primary aim of the study, regression-based moderation analysis was performed to test whether attitude toward sport plays a moderating role in the relationship between attitude toward violence and attitude toward sexual harassment. In the analysis conducted using Hayes’ PROCESS Macro Model 1, it was examined whether the relationship between the independent variable and the dependent variable varied across different levels of the moderator variable (attitude toward sport). The significance of the interaction term and the increase in the explained variance resulting from the inclusion of the interaction term in the model (ΔR^2^) were reported to evaluate the effect size.

To enhance the robustness of the results, the bootstrap method with 5000 resamples was employed, and 95% confidence intervals were calculated. Confidence intervals that did not include zero were interpreted as an indication that the moderation effect was statistically significant. The level of significance was set at *p* < 0.05 for all analyses.

## 3. Findings

Prior to testing the primary research hypotheses, descriptive statistics (mean and standard deviation) for the study variables (Attitude toward Violence, Attitude toward Sexual Harassment, and Attitude toward Sport) and the bivariate relationships between these variables were examined using Pearson correlation analysis. The findings are presented in Table 3.

Analysis of Table 3 reveals a statistically significant and positive moderate correlation between Attitude toward Violence and Attitude toward Sexual Harassment (r = 0.418, *p* < 0.001). Accordingly, it can be stated that as individuals’ attitudes toward violence increase, their scores for attitudes toward sexual harassment also increase. On the other hand, at the bivariate level, the Attitude toward Sport variable does not show a statistically significant relationship with either Attitude toward Violence (r = −0.002, *p* > 0.05) or Attitude toward Sexual Harassment (r = −0.098, *p* > 0.05).

However, the social science literature emphasizes that relationships between variables are generally not limited to simple and linear structures; instead, they may vary under different conditions and in the presence of third variables. In this context, examining moderation models provides a crucial analytical approach to identifying the conditions under which the relationship between variables strengthens or weakens (Gürbüz, 2021). Indeed, in multivariate moderation models, significant interaction effects can emerge depending on specific conditions, even in the absence of a direct (bivariate) correlation between the variables. Therefore, a moderation analysis was conducted in the subsequent stage to determine how the Attitude toward Sport creates a buffering or reinforcing condition within this network of relationships.

To examine the moderating role of Attitude toward Sport in the predictive effect of Attitude toward Violence on Attitude toward Sexual Harassment, a moderation analysis was conducted using the PROCESS Macro (Version 4.2, Model 1) developed by Hayes (2017). To control for potential heteroscedasticity, the HC3 (Heteroscedasticity-Consistent) standard error estimator was utilized. Additionally, to prevent multicollinearity issues, the independent and moderating variables were mean-centered prior to the analysis. Gender (1 = Male, 2 = Female) was included in the model as a control variable (covariate).

The overall moderation model was statistically significant, and when the independent variable, moderator, interaction term, and control variable were considered together, they accounted for 26.06% of the total variance in Attitude toward Sexual Harassment (R^2^ = 0.2606, F(4, 345) = 33.6073, *p* < 0.001). The mean squared error (MSE) of the model was calculated as 120.5327. The coefficient values and significance levels for the analysis are presented in Table 4.

When the main effects in Table 4 were examined, it was found that Attitude toward Violence positively and significantly predicted Attitude toward Sexual Harassment (β = 0.2707, t = 6.2812, *p* < 0.001), while Attitude toward Sport negatively and significantly predicted it (β = −0.1988, t = −3.9089, *p* < 0.001). The interaction term “Attitude toward Violence × Attitude toward Sport,” which is the primary focus of the research, was found to be statistically significant (β = −0.0098, t = −3.4010, *p* = 0.0008). This finding indicates that the interaction term was statistically significant, suggesting that the strength of the association between attitude toward violence and attitude toward sexual harassment varied as a function of composite sport attitude scores.

The gender factor, included as a control variable, also had a statistically significant effect on Attitude toward Sexual Harassment (β = 7.2583, t = 5.3955, *p* < 0.001). Examination of the coefficient values indicates that male participants (coded as 1 in this context) had significantly higher scores for attitudes toward sexual harassment compared to female participants. The inclusion of the gender variable did not weaken the interaction power between violence and sport attitudes; rather, it increased the explanatory power of the model and indicated that the conditional association remained statistically significant after controlling for gender.

To further examine the nature and direction of the identified moderating effect, a simple slopes analysis was performed at different levels of Attitude toward Sport (−1 Standard Deviation, Mean, and +1 Standard Deviation). The results are presented in Table 5.

Analysis of the conditional effect results in Table 5 reveals that the effect of Attitude toward Violence on Attitude toward Sexual Harassment is positive and significant across all levels (low, mean, high) of Attitude toward Sport. However, the magnitude of this effect varies depending on the level of sport attitude. When individuals’ attitudes toward sport are at a low level (−1 SD), the predictive effect of violence attitude on sexual harassment attitude is strong (Effect = 0.3861, *p* < 0.001). As sport attitude reaches the mean level, this effect decreases (Effect = 0.2707, *p* < 0.001). When individuals’ attitudes toward sport reach a high level (+1 SD), the predictive power of violence attitude on sexual harassment attitude drops to its weakest point (Effect = 0.1554, *p* = 0.0054).

Additionally, the Johnson-Neyman technique was applied, demonstrating that there is no transition point (point of non-significance) within the observed range of the data. The effect remained statistically significant throughout the entire spectrum, from the lowest observed sport attitude score (−37.8143; Effect = 0.6401) to the highest score (14.1857; Effect = 0.1321).

These findings indicate that the association between attitude toward violence and attitude toward sexual harassment was weaker at higher levels of the sport attitude composite. This pattern of conditional attenuation was statistically significant across all observed levels of the moderator.

**Figure 2:**
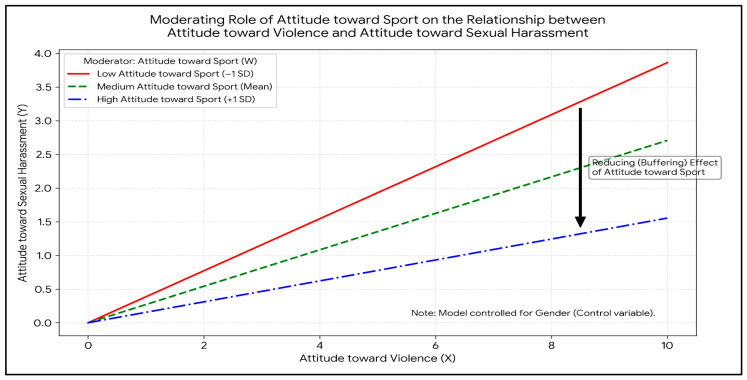
Interaction Effect of Attitude toward Violence and Attitude toward Sport on Attitude toward Sexual Harassment.

The simple slopes plot presented in Figure 2 visualizes the nature of the moderating effect of Attitude toward Sport. The graph indicates a positive relationship between Attitude toward Violence and Attitude toward Sexual Harassment across all levels of the moderator. However, as scores for Attitude toward Sport increase (indicated by the blue dashed line), the predictive power (slope) of violence attitude on sexual harassment attitude significantly decreases. The association between attitude toward violence and attitude toward sexual harassment was stronger at lower levels of sport attitude and weaker at higher levels, consistent with the pattern of statistical moderation observed in the regression model.

## 4. Discussion

The present study examined whether attitude toward sport moderates the association between attitude toward violence and attitude toward sexual harassment within a university sample. Consistent with the proposed hypothesis, attitude toward violence was positively associated with more accepting attitudes toward sexual harassment. In addition, the composite sport attitude scores significantly moderated this association. Specifically, the positive relationship between violence-supportive attitudes and harassment-supportive attitudes was weaker at higher levels of the sport attitude composite. However, the association did not disappear entirely across the observed range of the moderator variable, indicating a conditional attenuation—rather than elimination—of the association.

The positive association between violence-supportive attitudes and acceptance of sexual harassment aligns with prior gender-based and social-psychological research indicating that cognitive schemas legitimizing aggression are often intertwined with beliefs that normalize coercive or sexually exploitative behaviors (Bogen et al., 2022; Vescio et al., 2025). Research has shown that peer norms approving aggression and rape myth acceptance frequently co-occur, reflecting broader belief systems in which aggression and coercion are cognitively justified (Bogen et al., 2022). Experimental findings further demonstrate that masculinity threat and anger responses may increase favorable evaluations of sexual violence (Vescio et al., 2025). In this context, the present findings support the interpretation that harassment-supportive attitudes are closely aligned with broader violence-supportive orientations.

The statistically significant interaction term indicates that the strength of the association between violence-supportive and harassment-supportive attitudes varied as a function of sport attitude scores. Importantly, zero-order correlations indicated that attitude toward sport was not directly associated with attitudes toward violence or sexual harassment. Although the zero-order association was non-significant, the regression results indicated a small but significant negative main effect of sport attitudes when controlling for violence attitudes. This pattern may reflect a suppressor-like dynamic in which the association between sport attitudes and the outcome becomes apparent only within the full model structure (Hayes, 2017). However, this explanation is post hoc and should be interpreted cautiously. The absence of meaningful bivariate associations involving sport attitudes raises questions about the stability and substantive importance of the interaction effect. Replication in independent samples is necessary before drawing conclusions about the reliability of this pattern.

At the same time, prior research has documented contexts in which sport environments are associated with gender-inequitable norms, aggression-supportive peer climates, and power asymmetries—particularly in coach–athlete relationships—that may be associated with greater risk (Forbes et al., 2006; Bogen et al., 2022; Gaedicke et al., 2021; Verhelle et al., 2022). The present findings do not speak to these environmental factors directly, as the study measured self-reported attitudinal orientations rather than features of specific sport contexts. It should be explicitly noted, however, that the cross-sectional design of the present study does not permit directional or causal conclusions. The observed associations reflect statistical relationships within a theoretical framework, and reverse relationships—for instance, in which harassment-supportive attitudes shape violence-related evaluations—or the influence of unmeasured ideological factors cannot be ruled out.

The observed conditional association is noted in the context of Social Learning Theory (Bandura, 1977); however, the cross-sectional design does not permit conclusions about whether any learning or value internalization process underlies the statistical pattern. However, given the cross-sectional design, it is not possible to determine whether such a process underlies the observed statistical pattern. Research has shown that sportsmanship norms and climates of personal and social responsibility are associated with lower aggression levels (Courel-Ibáñez et al., 2019), and that team identification processes can either restrain or reinforce aggression-supportive narratives depending on how they are structured (Rudd & Stokowski, 2024). From this perspective, the observed attenuation may be theoretically consistent with such value-based processes; however, it should be noted that the sport attitude composite used in this study reflects a broad attitudinal disposition toward sport—encompassing moral, competitive, and social dimensions—rather than a narrowly defined moral-value orientation. As such, the specific mechanism underlying the observed statistical pattern cannot be identified from the present data.

The inclusion of gender as a control variable yielded a statistically significant effect on attitudes toward sexual harassment (β = 7.258, SE = 1.345, t = 5.396, *p* < 0.001), with male participants reporting significantly higher tolerance toward sexual harassment compared to female participants. This finding is consistent with a well-established body of research demonstrating that gender is among the most robust predictors of harassment-related attitudes, with men generally endorsing more permissive evaluations of harassment behaviors than women (Trottier et al., 2025; Sakallı-Uğurlu et al., 2010). The gendered nature of harassment-supportive beliefs likely reflects broader patterns of socialization in which traditional masculinity norms, rape myth acceptance, and adversarial sexual beliefs are more prevalent among male populations (Vescio et al., 2025; Bogen et al., 2022). Importantly, the inclusion of gender did not attenuate the core moderation effect; rather, the interaction term became more statistically robust (β = −0.010, *p* = 0.0008) and the overall model explained a substantially greater proportion of variance in harassment attitudes (R^2^ = 0.261 vs. 0.195). This pattern suggests that the conditional association was not fully explained by gender differences alone. However, it should be noted that gender represents only one of several potentially relevant covariates; variables such as sexism, gender role beliefs, prior sport participation, and exposure to specific sport environments were not assessed and may account for part of the observed association.

Although the moderation effect was statistically significant, the incremental variance explained by the interaction term was modest (ΔR^2^ = 0.011 in the original model). The interaction remained statistically significant after controlling for gender (β = −0.010, *p* = 0.0008); however, this does not fully address potential omitted variable bias, and the magnitude of the effect remains small. Given the small effect size and the absence of bivariate associations involving the moderator, the present findings should be treated as tentative and exploratory rather than as establishing a clearly interpretable moderation mechanism. The observed interaction identifies a statistical pattern that may inform future hypothesis generation, but does not, on its own, constitute strong evidence for a theoretically meaningful boundary condition. Replication using more targeted measures and stronger designs is required before substantive conclusions can be drawn.

Taken together, the findings indicate a statistically significant conditional association: the relationship between violence-supportive and harassment-supportive attitudes was weaker at higher levels of the sport attitude composite score. However, given that this composite aggregates across moral, competitive, and social dimensions of sport attitudes, it remains unclear which aspect of the broader sport attitude disposition—if any specific one—underlies this pattern. It should be emphasized, however, that this interpretation is based on self-reported value orientations rather than observed sport environments or climates. Whether similar patterns of conditional attenuation would emerge in relation to actual sport participation measures or specific sport contexts remains an open empirical question.

## 5. Conclusions

The present study found a positive association between attitudes toward violence and acceptance of sexual harassment among university students. In addition, the composite sport attitude score was associated with a statistically significant, though modest, conditional attenuation of this relationship.

These findings suggest that sport-related attitudinal orientations may be relevant to the study of harassment-supportive belief systems, and that future research examining the conditional structure of these relationships may be warranted. However, given the cross-sectional design, the modest effect size, and the broad nature of the sport attitude composite, the present findings should be regarded as preliminary and exploratory. The study contributes a statistical observation that may inform future hypothesis-driven research, rather than establishing a clearly interpretable or practically actionable conclusion.

## 6. Recommendations

The present findings are cross-sectional and based on self-reported attitudinal data; as such, they do not support direct practical prescriptions. The observed statistical patterns are, however, consistent with prior literature in which sport-related attitudinal orientations have been examined in relation to broader social belief systems (Verhelle et al., 2022; Courel-Ibáñez et al., 2019). These considerations may inform the design of future studies examining the relationship between sport-related attitudes and harassment-related beliefs across different populations and contexts.

Prior research has identified masculinity threat and aggression-supportive ideologies as relevant factors in the formation of harassment-supportive attitudes (Vescio et al., 2025; Gaedicke et al., 2021). Future research may examine whether these factors interact with sport-related attitudinal orientations in shaping harassment-related evaluations, particularly within coach–athlete relationships where power asymmetries are pronounced.

Intervention research has documented the effectiveness of interactive training models in improving harassment-related knowledge and prevention intentions (Cronin et al., 2024; McQueen & Murphy-Oikonen, 2025). Whether such interventions interact with sport-related attitudinal orientations remains an open empirical question that future research may address.

## 7. Limitations

Several limitations should be considered. First, the cross-sectional design prevents causal conclusions regarding the directionality of the observed relationships. Although moderation analysis identifies conditional associations, longitudinal or experimental designs are required to establish causal mechanisms.

Second, all measures relied on self-report instruments. Despite efforts to ensure anonymity and reduce social desirability bias, responses related to violence and sexual harassment may be influenced by impression management. Furthermore, the voluntary nature of participation introduces the possibility of self-selection bias: individuals with particularly strong or particularly moderate attitudes toward the constructs measured may have been more or less likely to participate, potentially affecting both the distribution of responses and the observed relationships among variables.

Third, the sample consisted of university students from a single institutional context. As prior research indicates that sport cultures vary substantially in their normative climates (Forbes et al., 2006; Gaedicke et al., 2021), the generalizability of the findings to other sport disciplines, competitive levels, or sociocultural environments may be limited.

Fourth, the present model included gender as a control variable; however, other potential covariates—such as sport participation history, athletic identity, or related psychosocial variables—were not examined. The inclusion of such variables in future research would provide a more comprehensive account of the attitudinal associations observed here.

Fifth, the Attitudes Toward Sport Scale yields a composite score aggregating across three subscales: ethical–moral values, competition, and social dimension. The present study examined the overall moderating role of sport attitudes without disaggregating these subscales. It is possible that different dimensions of sport attitudes exert differential moderating effects—for instance, ethical–moral value orientations may function as stronger buffers than competition-oriented attitudes. Future research should examine the moderating contributions of individual subscales to provide a more nuanced understanding of the mechanisms involved. More broadly, the construct validity of the composite sport attitude score is constrained by the heterogeneity of its subscale content: while the ethical–moral values subscale reflects normative moral orientations most directly relevant to the theoretical framework, the competition and social dimension subscales encompass content that is less proximally linked to value-based moral reasoning. Future research should examine the moderating contributions of individual subscales to determine which dimensions of sport attitudes are most theoretically and empirically relevant to the violence–harassment attitudinal link.

Finally, although statistically significant, the moderating effect size was modest. Therefore, practical significance should be interpreted with caution. Future research may investigate additional contextual or identity-based factors that interact with violence-supportive attitudes in shaping harassment-related evaluations.

## Figures and Tables

**Table 1:** Descriptive Statistics for the Gender and Age Distributions of the Participants (N = 350).

Variable	Category	n	%	x	SD
Gender	Female	158	45.1		
	Male	192	54.9		
Age				21.81	2.57

**Table 2:** Model Fit Indices and Psychometric Properties of the Measurement Models.

Index	Good Fit	Acceptable Fit	ATSH	ATV	ATS
χ^2^/df	≤3	3 < χ^2^/df ≤ 5	3.18	3.55	3.38
GFI	≥0.95	≥0.85	0.86	0.89	0.85
RMSEA	≤0.05	≤0.08	0.08	0.08	0.08
Cronbach’s Alpha	0.70 ≤ α ≤ 0.90	0.60 ≤ α < 0.70	0.70	0.841	0.932
Skewness	−1 to +1	−2 to +2	0.632	0.449	−0.532
Kurtosis	−1 to +1	−2 to +2	−0.089	0.356	−0.402

**Table 3:** Descriptive Statistics and Correlation Matrix for Research Variables.

Variable	x	SD	1	2	3
1. Attitude toward Violence	53.95	14.85	1		
2. Attitude toward Sexual Harassment	51.56	12.69	0.418 **	1	
3. Attitude toward Sport	95.81	11.81	−0.002	−0.098	1

Note: M = Mean, SD = Standard Deviation. N = 350. ** *p* < 0.001.

**Table 4:** Model Coefficients Regarding the Moderating Role of Attitude toward Sport.

Variables	Coefficient (β)	SE (HC3)	t	*p*	%95 CI (LL)	%95 CI (UL)
Constant	40.3132	2.0742	19.4354	<0.001	36.2335	44.3929
Attitude toward Violence (X)	0.2707	0.0431	6.2812	<0.001	0.1859	0.3555
Attitude toward Sport (W)	−0.1988	0.0508	−3.9089	<0.001	−0.2988	−0.0987
Interaction (X × W)	−0.0098	0.0029	−3.4010	0.0008	−0.0154	−0.0041
Gender (Control)	7.2583	1.3453	5.3955	<0.001	4.6124	9.9043

Note: SE = Standard Error, CI = Confidence Interval, LL = Lower Limit, UL = Upper Limit. Independent and moderating variables are mean-centered. Gender coded as 1 = Male, 2 = Female.

**Table 5:** Conditional Effects of Attitude toward Violence at Different Levels of Attitude toward Sport.

Level of Attitude Toward Sport	Value	Effect (Coeff)	SE (HC3)	t	*p*	%95 CI (LL)	%95 CI (UL)
Low (−1 SD)	−11.8063	0.3861	0.0542	7.1282	<0.001	0.2795	0.4926
Mean	0.0000	0.2707	0.0431	6.2812	<0.001	0.1859	0.3555
High (+1 SD)	11.8063	0.1554	0.0555	2.7984	0.0054	0.0462	0.2646

Note: SD = Standard Deviation. “Effect” refers to the predictive coefficient of Attitude toward Violence on Attitude toward Sexual Harassment.

## Data Availability

The datasets generated and analyzed during the current study are available from the corresponding author on reasonable request.
